# Unveiling Nanoscale Heterogeneities at the Bias-Dependent Gold–Electrolyte Interface

**DOI:** 10.1021/jacs.3c11696

**Published:** 2024-04-09

**Authors:** Leo Sahaya Daphne Antony, Loriane Monin, Mark Aarts, Esther Alarcon-Llado

**Affiliations:** † AMOLF, Amsterdam 1098 XG, The Netherlands; ‡ Leiden Institute of Chemistry, Leiden University, Leiden 2333 CC, The Netherlands; § Van’t Hoff Institute for Molecular Sciences, University of Amsterdam, Amsterdam 1090, GD, The Netherlands

## Abstract

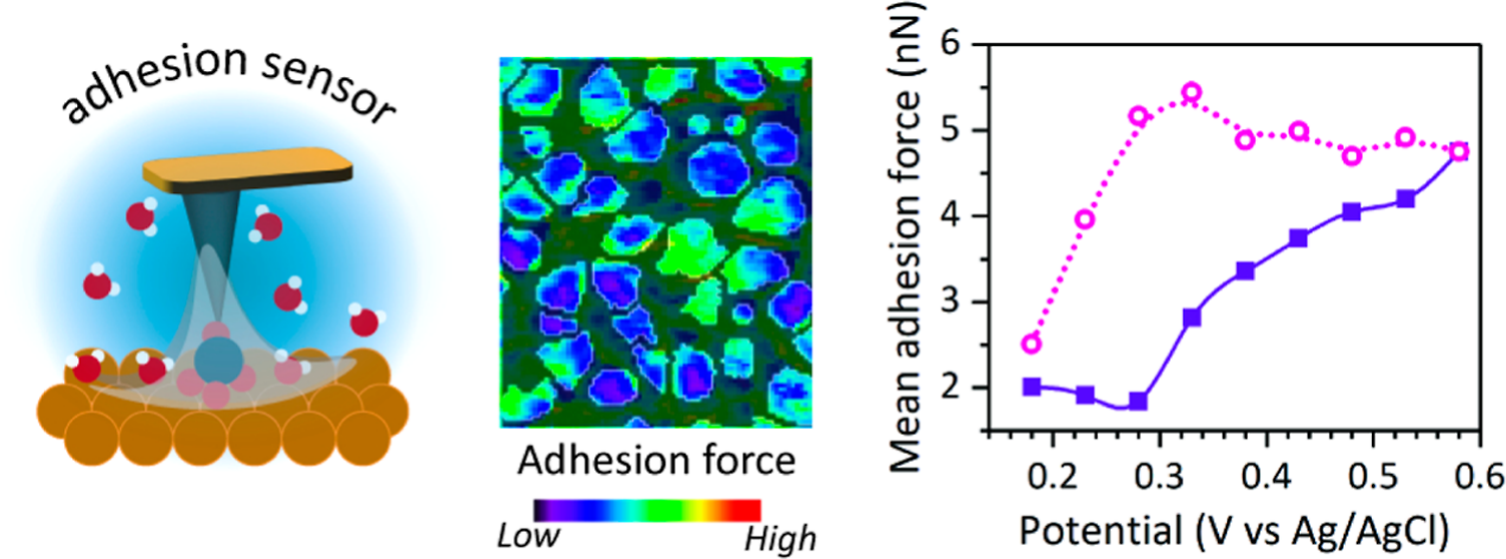

Electrified solid–liquid interfaces (SLIs) are extremely complex and dynamic, affecting both the dynamics and selectivity of reaction pathways at electrochemical interfaces. Enabling access to the structure and arrangement of interfacial water in situ with nanoscale resolution is essential to develop efficient electrocatalysts. Here, we probe the SLI energy of a polycrystalline Au(111) electrode in a neutral aqueous electrolyte through in situ electrochemical atomic force microscopy. We acquire potential-dependent maps of the local interfacial adhesion forces, which we associate with the formation energy of the electric double layer. We observe nanoscale inhomogeneities of interfacial adhesion force across the entire map area, indicating local differences in the ordering of the solvent/ions at the interface. Anion adsorption has a clear influence on the observed interfacial adhesion forces. Strikingly, the adhesion forces exhibit potential-dependent hysteresis, which depends on the local gold grain curvature. Our findings on a model electrode extend the use of scanning probe microscopy to gain insights into the local molecular arrangement of the SLI in situ, which can be extended to other electrocatalysts.

## Introduction

Full control over the solid–electrolyte or solid–liquid interface (SLI) is central to solving the renewable energy conversion and storage problem. The local structure and dynamics of the potential-dependent SLI at the electrocatalyst determines the electrochemical performance in electrolyzers, fuel cells, and batteries as the SLI properties govern processes such as electron transfer kinetics, ion adsorption/desorption, and reaction overpotentials.^1−4^

Many efforts have focused on design principles and activity descriptors of catalysts, where the covalent adsorbate–solid interaction is tuned through modulating the (surface) electronic structure.^5^ More recently, it has been noted that the chemico-physical property of electrolytes (such as noncovalent interactions and solvation environments at the electrified interface) is an equally important parameter that significantly impacts catalytic activity and selectivity.^6−8^ In particular, it has become apparent that the catalyst–electrolyte interface dynamically adapts to the operating conditions, where both the solid catalyst and the electrolyte have equally important roles.^3,9^ On the liquid side, the electrolyte composition (pH, ionic species) has a direct impact on surface and interface properties through (de)protonation, ion adsorption/intercalation, etc. The interfacial electrolyte structure controls the transport of reactants and products to, from, and at the surface and affects the density of key reaction intermediates.^10,11^ Rational designing of next-generation electrocatalysts thus requires a complete nanoscale description of the SLI and its evolution with electrochemical parameters,^12^ the most important one being the external bias potential.

In water-based electrolytes, several in situ and *operando* techniques, including vibrational spectroscopy (Raman,^13−15^ FTIR,^16,17^ Second Harmonic Generation^18,19^), X-ray-based methods^20,21^ (XPS, XAFS), force-based methods (surface force apparatus (SFA)^22,23^ atomic force microscopy (AFM)^24−27^), have been employed to access various aspects of the dynamic interfacial water structure under external bias. Among these, electrochemical AFM offers in situ/*operando* correlative measurements that can probe both the liquid and the solid with nanometer spatial resolution, providing access to nanoscale heterogeneities. Surface morphology together with local conductivity,^28^ electrochemical activity,^29,30^ and more have revealed the existence of nanoscale catalytic hot spots on apparently homogeneous surfaces. Similar to the electrochemical SFA,^31,32^ the force measurement in electrochemical AFM contains abundant information on the SLI structure with nanoscale to atomic resolution.^22,33−41^

Earlier works on bias-dependent interfacial energy measurements ignore the thermodynamic influence on the SLI and often provide contradicting interpretations as to what influences the observed forces at the SLI, such as hydrogen bonding between tip-electrode,^37^ surface tension,^42^ or electrostatic influence between the tip-electrode.^39^ Furthermore, to the best of our knowledge, there are no existing works that provide spatial maps of the interfacial forces on electrode surfaces as a function of bias potential.

In this work, we use peak-force electrochemical AFM (EC-AFM) for the concurrent mapping and tracking of topography and interfacial adhesion forces on nanostructured gold film electrodes as a function of bias. The measured adhesion force interpreted as the interfacial energy of the SLI^34^ is directly linked to the thermodynamic work to reorganize the interface. This in turn has direct implications for the activation energy and other electrochemical processes. Our in situ force measurements enable the visualization of spatial force heterogeneities across the electrode/electrolyte interface irrespective of the applied bias potential. By studying the potential-dependent response, we observe the contribution of solvent and anions to the interfacial adhesion forces. This work demonstrates the use of AFM adhesion force measurements to study the local differences in the SLI on a model (111)-oriented polycrystalline gold electrode under varying electrochemical conditions. We observe a marked dependence of the behavior of the SLI as a function of the local curvature of single grains, smaller than 100 nm. Furthermore, we observe inhomogeneities at the single grain level, characterized by two distinct surface states that exhibit similar potential-dependent responses. Our work implies further results in local differences in ion/solvent ordering^43^ and viscosity of the interfacial layer. Due to the generality and high resolution of electrochemical AFM, our methodology can readily be extended to additional model electrocatalysts or nanoparticle systems.

## Results and Discussion

In this work, we investigate the SLI formation energy at (111)-oriented polycrystalline-gold electrodes in a 10 mM *Na*_2_*SO*_4_ aqueous solution at the potential window of the electric double layer (EDL) formation with specific anion adsorption^44,45^ (0.18–0.58 V vs Ag/AgCl). The potential window was specifically selected to avoid faradaic processes like gold oxidation, and the maximum load of the Silicon Nitride tip was set to 10 nN (see SI Figure S1). All potentials in this work are given with respect to a Ag/AgCl reference, unless specified otherwise.

**Figure 1:**
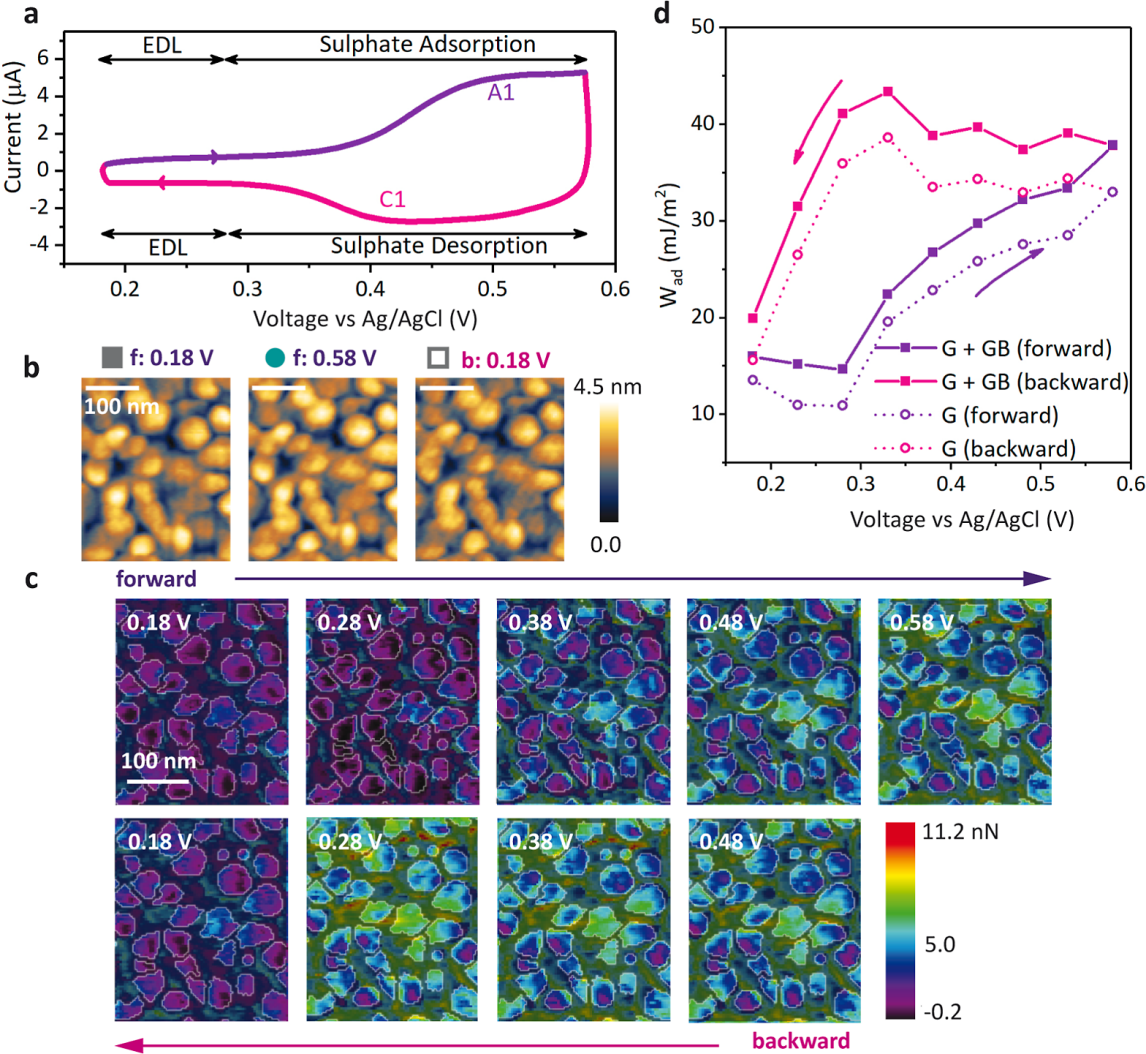
(a) CV reading recorded on the gold electrode red in a 10 mM *Na*_2_*SO*_4_ electrolyte. (b) Topography maps of the same area at three different potentials, from left to right: 0.18 (forward scan), 0.58 (forward scan), and 0.18 V (backward scan). Scale bar: 100 nm. (c) Adhesion force maps of the same area as shown in (b) acquired during stepwise potential bias at the gold electrode in a 10 mM*Na*_2_*SO*_4_ electrolyte. Scale bar: 100 nm. (d) Mean thermodynamic work of adhesion derived from the measured adhesion force map as a function of potential (DMT contact mechanics model), with (G + GB) and without (G) including the grain boundaries.

The typical cyclic voltammetry (CV) measurement of the gold electrode is plotted in Figure 1a. The forward (or anodic) sweep shows an initial capacitive EDL charging (∼0.18–0.35 V), which is followed by an anodic wave (labeled A1) related to the adsorption of sulfate anion on the gold surface.^46^ In the backward (or cathodic) scan, the reversible processes occur, leaving the electrode devoid of sulfate ions at the end of the cathodic wave (∼0.32 V, labeled C1) followed by the EDL discharge. Note that in CV reading, we do not observe a clear peak related to the lifting of the Au(111)( × 23) surface reconstruction in freshly made samples,^47,48^ which is not unusual in polycrystalline films or crystals with small (111) terraces.^46^

With the aim of probing the voltage-dependent electrode–electrolyte interface structure with nanometer spatial resolution, we simultaneously image the topography and local adhesion in situ with peak-force EC-AFM as the potential is swept stepwise from 0.18 to 0.58 V and back. The SLI formation energy is probed at every tapping cycle by measuring the amount of work that is needed to separate the tip from the electrode (known as the thermodynamic work of adhesion, *W*_adh_(49)). This work includes the amount of energy that is needed to reform the SLI at the electrode and at the tip upon separation. At the same time, *W*_adh_ is related to the associated interfacial energies of the broken and formed interfaces by the Young-Dupré equation
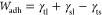
1where γ_tl_, γ_sl_, and γ_ts_ are the interfacial energies of the tip–liquid, sample–liquid, and tip–sample, respectively. In AFM, the local force of adhesion (*F*_adh_) between the tip and the sample is measured. According to the theory of contact mechanics, *F*_adh_ can be given as
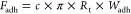
2where *W*_adh_ is the work of adhesion, *R*_t_ is the tip apex radius (22.97 ± 0.085 nm), and the proportionality constant, *c* = 2 or 1.5, for Derjaguin, Muller, and Toporov (DMT) and Johnson–Kendall–Roberts adhesion models, respectively. The elastic properties of the tip and substrate determine the model one should use.^50^ In this work, we apply the DMT adhesion model. As we do not observe significant changes in the tip radius throughout the course of the experiment (based on topography imaging quality, scanning electron microscopy and blind tip estimation, see Methods and SI), we use *F*_adh_ and *W*_adh_ interchangeably for the rest of the article.

Figure 1b shows in situ topography images of the same area of the gold electrode taken at three of the potentials (0.18, 0.58, and back to 0.18 V). All the topography images at other different applied potentials are shown in SI Figure S5. The topography images reveal the presence of small (<100 nm wide, <10 nm protruding) grains that are mostly oriented in the (111) direction (SI Figure S6). The great similarity between the three topography maps indicates that there are no substantial changes in the sample morphology upon applying the potential bias (see a more detailed analysis and cross-sectional profiles in the SI Figure S7). Yet, upon closer look, we have consistently noticed a slight (<0.9 nm) increase in grain height during the adsorption and desorption of anions. While we cannot exclude a possible systematic error in the z-resolution during the measurement, we also note that the mobility of gold adatoms is expected to be restricted by the presence of multi bonded anions like sulfates and phosphates.^51^ To identify the grains, we have used the topography map at the lowest potential to highlight the grains through a semitransparent mask. The same mask has been applied to the rest of the images upon drift correction (see the Supporting Information and Figure S8 for more details on grain identification).

Figure 1c shows adhesion maps of the same area obtained at every other applied potential, for which the mean value of work of adhesion in each map is shown in Figure 1d (solid markers). The experimental error associated with the adhesion force measurement is estimated to be <0.1 nN (see SI Section 32). We have used the same color scale range for all maps and applied the semitransparent outline for easy comparison. The adhesion maps without the mask can be found in Figure S11. The maps clearly show an inhomogeneous distribution of the adhesion force across the surface that collectively increases as the potential rises from ≳0.3 V. In the backward scan, the adhesion maps remain virtually unchanged until the adhesion force drastically decreases for potentials <0.28 V, leaving a very similar adhesion map at the end and beginning of the potential sweep. We also note that the adhesion is consistently lower at the grains compared to grain boundaries. The higher adhesion force measured along some grain boundaries is likely attributed to either multiple contact areas in narrow trenches at adjacent grains (i.e., leading to an increased effective tip radius) or to the different surface properties at grain boundaries.

A more quantitative representation of the overall (or macroscopic) trend is given by the mean work of adhesion, ⟨*W*_adh_⟩ (Figure 1d), which is obtained from the mean value of the adhesion force using the DMT model. In addition to the average from the whole scan area (filled squares, Figure 1d), we also considered pixels only inside the grains (open circles, Figure 1d) identified by the mask. Neglecting the grain boundaries in ⟨*W*_adh_⟩ does not change the overall trend with voltage but mostly shows an offset with respect to the values for the whole scan area.

While several interactions contribute to the absolute value of *W*_adh_, we expect that changes in *W*_adh_ with varying potential mainly arise from the bias-dependent SLI structure at the gold electrode (i.e., Δ*W*_adh_ ≈ Δγ_sl_(V)) and can therefore be correlated to electrode processes in the CV. We assume a minimal contribution from Δγ_ts_(V) at the given experimental conditions due to negligible surface charges on the tip due to the isoelectric point of the tip.^38^ In the case of using a charged tip, additional tip–sample electrostatic attraction should be taken into account.^23^ However, in our experimental pH conditions, the tip is close to its isoelectric point, which minimizes tip–sample electrostatic effects.

As indicated by Figure 1c, ⟨*W*_adh_⟩ is small in the double layer region of the forward scan (purple curves), and it shows a shallow minimum at ≈0.28 V, which is close to the electrode’s point of zero charge (PZC ≈ 0.23 V vs Ag/AgCl, see SI). A minimum of the adhesion work around the PZC is not unexpected as the EDL formation energy is minimized close to the PZC. At potentials negative or positive from the PZC, γ_sl_ (and therefore the ⟨*W*_adh_⟩) is expected to increase with voltage due to the reorienting of water molecules and the decreasing configurational entropy of the solvent by the electrode charge-induced field and increased ion migration from the bulk solution to the interface to screen the electrode charge (i.e., electrostatic charging). This explains the rise in ⟨*W*_adh_⟩ for *V* < 0.28 and *V* > 0.28 V.

In the presence of anion adsorption, Schoenig et al.^45,52^ have shown that the (negative) reaction entropy levels off, reaching a shallow minimum at intermediate anion coverages. This effect may explain the reduction in the adhesion work increase rate once sulfate adsorption starts (≈0.38 V). Also, we suspect that the tip is only partially perturbing the SLI, where the diffuse layer and solvated water network are disrupted, but chemisorbed anions are not squeezed out. The partial charge compensation from chemisorbed anions would also result in a reduction in the adhesion work increase rate as anions are adsorbed. Previous works on macroscopic SFA studies on similar solid/liquid electrolyte systems are consistent with our observations.^22,23,37,39,53,54^

When the voltage cycle is reversed, we observe a clear hysteresis in the work of adhesion, which is not unusual in electrochemical processes. As the potential is reduced from high voltages, ⟨*W*_adh_⟩ remains stable at high values and shows a slight increase followed by a steep drop at voltages around the end of sulfate desorption (∼0.3 V in the reverse scan). At the end of the reverse scan, ⟨*W*_adh_⟩ reaches values similar to those at the beginning of the scan, indicating the reversibility of the process. Since the measurements here are done in the steady state, the hysteresis is not related to kinetic effects but rather means that the EDL structure at a given voltage depends on its bias history.

Taking advantage of the nanoscale nature of the AFM probe, we now explore the adhesion across the Au grains and potential correlations with properties from the topography data. First, we assess whether the observed spatial and/or temporal heterogeneities arise due to the influence of electrode grain morphology. For instance, film roughness can strongly affect adhesion forces due to changes in the asperity contact. In our case, the average surface roughness is ∼0.7 ± 0.06 nm (297 nm × 355 nm scan area) throughout all applied potentials. Therefore, we rule out roughness as the source of the observed spatial inhomogeneities in the adhesion force maps.

**Figure 2:**
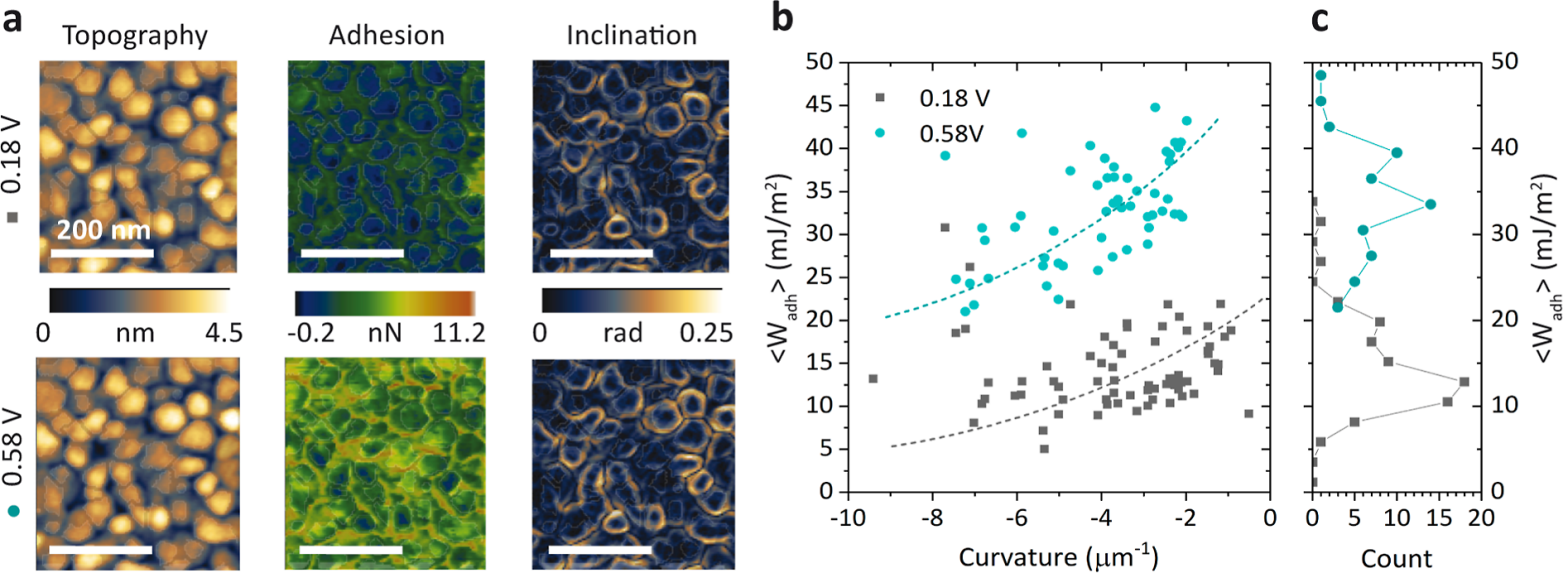
(a) Topography, adhesion, and inclination of the same area at the lowest (0.18 V, top) and highest applied potentials (0.58 V, bottom). (b) Mean grain adhesion work versus grain curvature of all grains in the adhesion maps shown in (a). The work of adhesion has been calculated by using the extended DMT theory, which includes grain curvature effects on the contact area.

On the other hand, local surface inclinations increase the tip contact radius, and consequently so does the adhesion force value. However, Figure 2a shows that the surface under study is consistently flat with potential (local inclinations up to 0.25 rads/14.32°, which is much smaller than the tip angle). We therefore conclude the influence of grain inclinations to be insignificant in our experiment.

Recently, Munz et al.^28^ suggested that grain curvature plays a role in determining the local electrical and mechanical properties of interfacial water at polycrystalline-metal electrodes under an applied bias based on frictional force and conductive studies. We have computed the curvature of all of the grains in the scan area and plotted them against the average work of adhesion within each grain for the lowest and highest potentials (Figure 2b). Here, negative curvature values indicate convex surfaces. To account for curvature effects on the force measurement, we use the extended expression that relates work of adhesion and adhesion force, given in the inset (see SI for more details). Figure 2b highlights a clear negative correlation between grain curvature and average adhesion, indicating a higher formation energy of the Au–liquid interface in flatter grains. The increasing work of adhesion as the grain curvature decreases may arise from increased intermolecular interactions at the SLI in these flatter grains.^55^ Such a tighter interfacial water network might explain the observed increase in friction forces at the SLI observed by Munz et al.^28^ An explanation for the lower adhesion at high curvature grains may arise from reduced structural order and density of interfacial water^43^ due to a high density of step edges and/or high order crystal facets.

It is also noteworthy to mention that at the lowest potential (0.18 V), most of the data points are centered around a similar low value of adhesion (see the histogram in Figure 2c, gray curve). In contrast, at the highest potential (0.58 V, Figure 2c, blue curve), the data point distribution becomes more uniform across a wider range. This is an indication that not all grains follow the same behavior with voltage.

**Figure 3:**
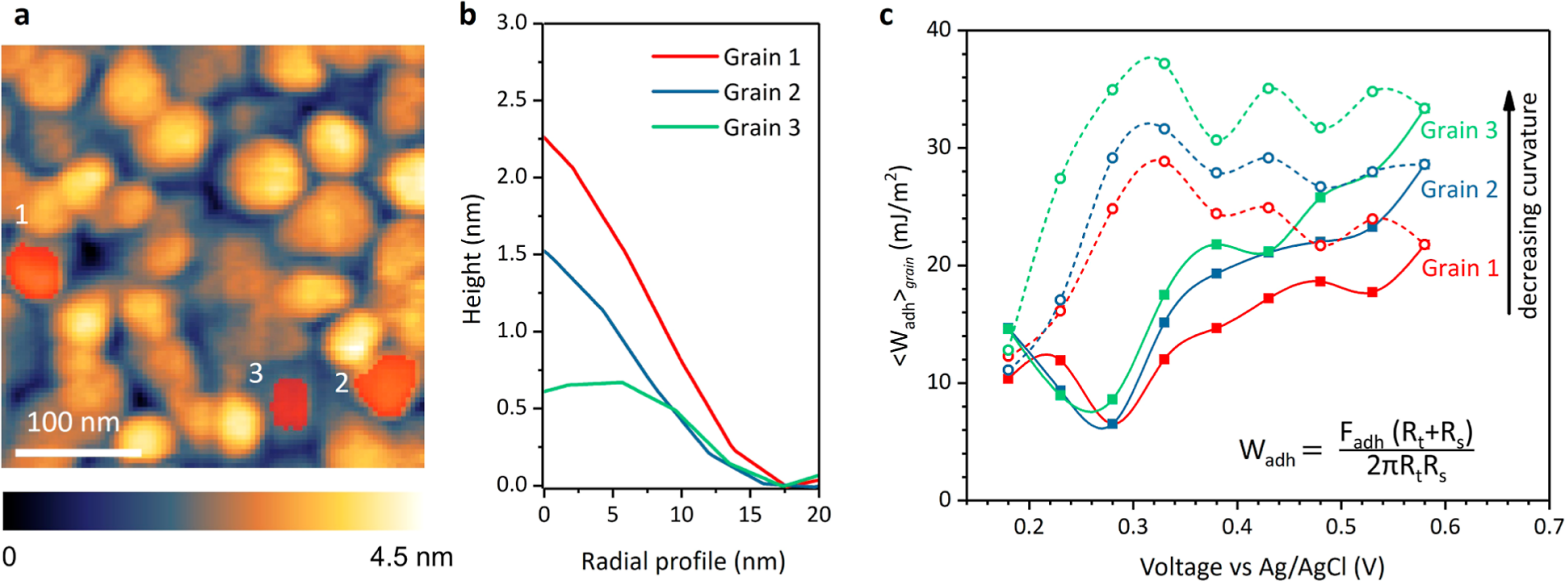
(a) Scan area with three marked grains with different curvatures (grain 1:8.9, grain 2:4.0, grain 3:2.8 μm^–1^). (b) Their corresponding radial profiles. (c) Bias-dependent work of adhesion of the three marked grains corrected with respect to their individual grain curvatures.

We further investigate the role of grain curvature in the bias-dependent adhesion by tracking the behavior of three arbitrarily selected grains with distinct curvatures as shown in Figure 3a. Figure 3b shows the radially averaged profiles of the marked grains. From 1 to 3, the grain curvature decreases as well as the total height. However, the grain inclinations remain similar (see SI Figure S16). As the grain curvature can influence the measured *F*_adh_ (see SI Figure S13, we calculate the ⟨*W*_adh_⟩) of the grains using the extended DMT theory that takes grain curvature into account as shown in Figure 3c. The mean adhesion force of each grain as a function of the applied potentials is shown in SI Figure S14a. While the ⟨*W*_adh_⟩ of the three grains follow a qualitative trend similar to that shown in Figure 1d irrespective of their curvature, the absolute increase in adhesion force with bias is strongly influenced by the curvature. We note that the ⟨*W*_adh_⟩ value near the PZC is similar for all grains, but the rate of increase in adhesion with *V* > PZC is enhanced in flatter grains, pointing again to the fact that the flatter the grain, the higher the order that is induced in the solvent/ionic arrangement at the interface. This trend is consistent throughout the grains in the scan area (see the SI section on Statistical analysis of grain curvature dependent adhesion forces.)

**Figure 4:**
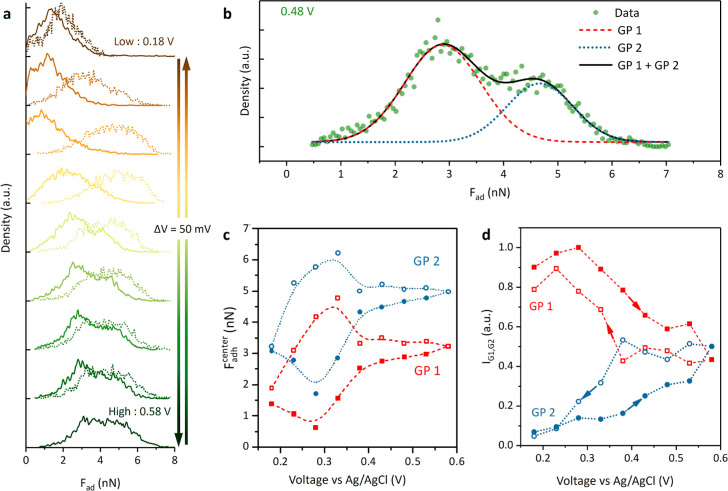
(a) Density distribution of adhesion forces in the maps from Figure 1. Forward and reverse voltage sweeps are shown as solid and dashed lines, respectively. (b) Example of the fitting of the density distribution with two Gaussian functions. The fitted center position and intensity of the two Gaussian are shown in (c) and (d), respectively.

Irrespective of intergrain analysis, we also note distinct adhesion force inhomogeneities within the marked grains (SI Figure S18). We further inspect the intragrain adhesion behavior by looking at the evolution of the distribution of adhesion forces with bias (Figure 4a). For this, we consider only those pixels marked as grains by the watershed algorithm. While the adhesion distribution at low bias appears to be described by a single broad peak, the histogram clearly evolves into a bimodal distribution at potentials corresponding to sulfate adsorption.

By fitting all distributions with a bigaussian function with unfixed distribution centers (see for instance the fit for the distribution at 0.48 V in Figure 4b), we extract the center position and intensity of each Gaussian (denoted as GP1 and GP2) as a function of potential (Figure 4c,d). Strikingly, we find that the central position of both Gaussians evolves in a parallel fashion with an almost constant difference of 1.65 ± 0.08 nN (7.59 mJ/m^2^) and 1.71 ± 0.11 nN (7.86 mJ/m^2^) in the forward/backward scans, respectively. The largest deviation is found at potentials around the onset of sulfate ad/desorption, where the fit is less accurate. The potential-dependent intensities of GP1 (low adhesion) and GP2 (high adhesion) reveal that most of the surface starts as GP1 at the beginning of the potential cycle, and it evolves into about 50:50 GP1:GP2 upon anion adsorption. The high adhesion regions can be considered as “hard to activate”, requiring higher thermodynamic work to reorganize the interface for other electrode processes. Upon reversing the cycle, the surface eventually goes back to being mostly GP1.

The constant difference of GP1 and GP2 central position and bias-dependent intensities points to the surface of gold having two energy states that locally switch from one another depending on the applied potential. The steady coevolution and hysteresis of GP1 and GP2 intensities with potential point to the fact of adsorbed anions having a key role in regulating the population distribution between the two surface states. We suspect these noticeable nanoscale differences in surface states could potentially originate from local differences in EDL due to uneven surface charge distribution through small differences in crystal orientations, resulting in uneven anion adsorption. These local heterogeneities lead to local differences in ion/solvent ordering^43^ and viscosity of the interfacial layer. The significance and nature of these two surface states should be further investigated to unravel their potential role in modulating the local catalytic activity.

## Conclusions

To summarize, we have demonstrated that AFM-based adhesion force mapping of electrified Au(111)–electrolyte interface is a powerful method to get nanoscale insights into the EDL formation energy. In this work, we show that adhesion forces are a way to qualitatively probe changes in interfacial water network ordering and stability, where higher adhesion force values indicate an increase in water ordering/density. We observe a clear hysteresis in the average adhesion force with potential, which is ruled by the specific adsorption of sulfate anions. From an exhaustive correlation between adhesion and nanoscale morphology, we find a distinct negative correlation between the average adhesion force value at a given grain and its curvature, irrespective of the applied potential. It appears that the high density of step edges and/or high-order crystal facets in high curvature grains disrupt the structural order and density of interfacial water. By tracking the adhesion in a few individual grains with distinct curvatures, we notice that grains with low curvature exhibit a larger hysteretic response with potential. A closer inspection on the subgrain distribution indicates that local inhomogeneities arise from the evolution of two distinct surface states, the population of which is modulated by the applied potential.

This work opens the door to future work probing the SLI in the presence of different types of (non)specifically adsorbing anions and cations as well as studying the dynamics of EDL formation via measuring adhesion forces at applied potential pulses.

## Materials and Methods

### Materials

For the preparation of the electrolyte, sodium sulfate (*Na*_2_*SO*_4_, ACS reagent ≥ 99.0%) salt purchased from Sigma-Aldrich was mixed with Millipore water. The pH of the electrolyte was measured to be 6.61 on the pH scale. The studied gold electrodes were prepared by e-beam deposition of gold on a clean (111) silicon wafer. The Si wafer was first coated with 5 nm thick titanium as the adhesion layer. 50 nm of gold was then deposited on top of the adhesion layer at the rate of 0.05 nm/s. The final roughness of the gold electrode was measured as 4.23 ± 1.05 nm (500 nm scan area). The gold electrodes after deposition showed columnar growth with predominately Au(111) terraces, as indicated by the X-ray diffractograms in the SI Figure S6.

### Adhesion Force Mapping Using Electrochemical-AFM

The adhesion force mapping was performed on a Bruker Dimension Icon AFM system equipped with a nanomechanical mapping module. The adhesion forces were measured using Silicon Nitride Scanasyst-Fluid tips (Bruker) of nominal radius = 20–60 nm, spring constant = 0.7 N/m, and frequency = 150 kHz. The probe was mounted on a special EC-AFM holder (TM) surrounded by a splash shield. The laser was aligned to get a sum of ≈3.4 V to ensure a robust feedback during the experiments. The drive frequency and the peak force amplitude for the *z*-axis modulation were set to 2 kHz and 100 nm, respectively. The deflection sensitivity of the tip was measured both in air and in the electrolyte before adhesion force mapping. The spring constant of the tip was obtained via thermal tuning estimation in the electrolyte. The tip apex radius was estimated to be 22.97 ± 0.08 nm (see SI section: Estimation of tip radius). This value is used throughout the article to calculate the work of adhesion values from the measured adhesion force values.

We perform the AFM measurements at (macroscopic) steady-state conditions, i.e., upon macroscopic current reaching the steady state after bias is applied. During imaging, the tip is only locally perturbing the double layer. Given the very low drive frequency of the tip (2 kHz), the double layer is expected to fully recover at each oscillation. For 10 mM *Na*_2_*SO*_4_ solution, τ_D_ is estimated to be about 2 ns, which is much smaller than the oscillation time of the probe (500 μs), ensuring a full recovery of the double layer after every contact. All the force mapping was done at the same maximum set point force of 10 nN (see the Supporting Information for more details). The experiments were conducted on a custom-built electrochemical cell (EC-Cell) with a three electrode configuration. The deposited gold electrodes, cut into a 4 × 4 cm^2^ area, acted as the working electrode (WE). A platinum wire around the WE and a leakless Ag/AgCl microelectrode served as the counter and reference electrodes, respectively. A freshly prepared 10 mM sodium sulfate aqueous solution acted as the electrolyte. All potentials reported in this work are calibrated vs the Ag/AgCl electrode (in a saturated KCl solution).

### Experiment Protocol

Before every experiment, the gold WE was rinsed thoroughly in water, acetone, and isopropanol and dried. The EC-cell components and the counter electrode were sonicated in Millipore water for 30 min before every experiment. The reference electrode was calibrated against a standard Mother electrode. CV is always performed first on the gold electrode. Next, a potential of −0.1 V vs Ag/AgCl is applied on the WE for 60 s as preconditioning. This is done to ensure that the polycrystalline WE electrodes are prepared in a similar structure before the adhesion mapping experiments. For the AFM experiments, the potentials are usually applied from low - high - low potentials based on the potential window range under study using the chronoamperometry technique. The AFM measurements were usually performed in the steady-state region of the chronoamperometry (30 s after the potential is applied). After the AFM measurements, a CV is done to see if there are any changes in the behavior of the electrode.

## Supporting Information Available

Maximum set point for adhesion force mapping, estimation of tip radius, estimation of experimental error, topography images at different applied potentials, structural characterization, cross-sectional profiles of topography images at three different applied potentials, grain identification, potential-dependent adhesion maps with no applied mask, the influence of surface grain curvature on the work of adhesion, potential-dependent adhesion forces of the marked grains, statistical analysis of grain curvature-dependent adhesion forces, inclination maps, estimation of PZC by the voltammetric approach, closeup of a single marked grain at three different applied potentials, and spatial distribution of GP1 and GP2 (PDF)
