# Supplementary material for: Unveiling Nanoscale Heterogeneities at the Bias-Dependent Gold–Electrolyte Interface
Source: J Am Chem Soc. 2024 Apr 9;146(19):12933–40. doi: 10.1021/jacs.3c11696 (PMC11099963; doi:10.1021/jacs.3c11696)
Supplement: Supplementary file 1 — ja3c11696_si_001.pdf [file ja3c11696_si_001.pdf]

Supporting Information: Unveiling nanoscale heterogeneities at the bias-dependent Gold-Electrolyte Interface

Leo Sahaya Daphne Antony,[†] Loriane Monin,[†] Mark Aarts,[‡] and Esther Alarcon-Llado[*,†,¶]

†*AMOLF, Amsterdam, Netherlands*

‡*Leiden University, Leiden, Netherlands*

¶*Vant Hoff Institute for Molecular Sciences, University of Amsterdam, Amsterdam, 1090 GD, The Netherlands*

E-mail: e.alarconllado@amolf.nl

1 Maximum setpoint for adhesion force mapping

The adhesion forces were mapped in real-time during the data acquisition via Nanoscope software. The spring constant of the used Silicon Nitride probes are 0.68-0.72 N/m with a nominal radius of 20-60 nm (based on the manufacture's description). In order to achieve a highly localised information on a poly-crystalline electrode, it was important to ensure a proper setpoint or maximum force on the cantilever, as described by previous works[2,3]. Adequate setpoint ensures proper contact of the tip on the surface, as the tip descends through the electric double layer. Additionally it enables sufficient deformation of the cantilever without inducing a severe indentation on the sample upon contact. To identify this setpoint, the oscillation amplitude of the cantilever was fixed to 100 nm and adhesion maps were acquired for various setpoints with and without the application of potential.

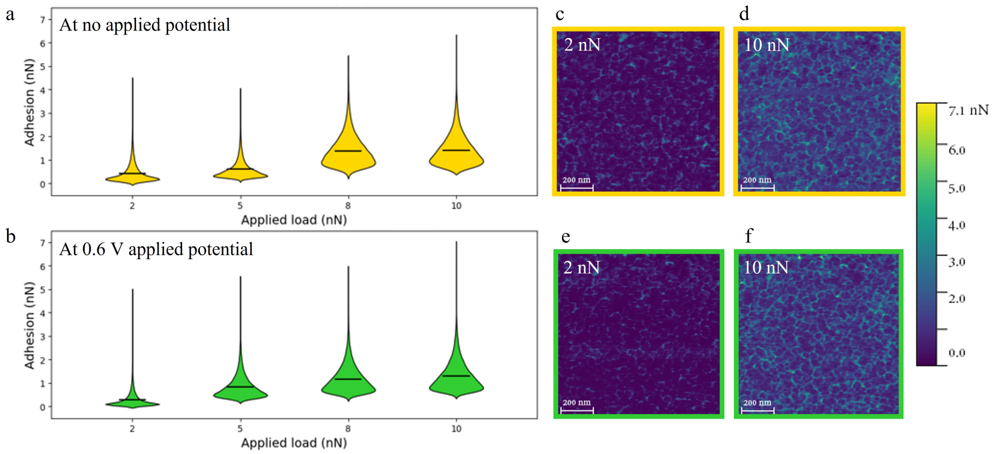

Figure S1: Adhesion distributions as a function of applied setpoint(load) (a)with no potential, (b)with applied potential of 0.6 V. Corresponding adhesion maps obtained for no potential at (c)2 nN and (d)10 nN setpoints respectively.Corresponding adhesion maps obtained for 0.6 V applied potential at (c)2 nN and (d)10 nN setpoints respectively.

Figures S1a and S1b show the adhesion force distribution of the scan area plotted against different setpoints (applied load) with no applied and 0.6V applied potentials respectively. In both cases, as the setpoint is increased the adhesion force distribution increases until it reaches a certain threshold (around 8 nN). Any further increase in setpoint (as 10 nN) results in similar distribution as the previous setpoint. When the adhesion maps of the same area with a low setpoint (2nN) and high setpoint (10nN) is compared, the maps of the high setpoint reveal more details along the grain boundaries. So, the setpoint was set to 10 nN in all the measurements reported in this work, to ensure similar acquisition of local adhesion force information.

2 Estimation of tip radius

Before the measurements are performed in the electrolyte under applied potentials, a blind tip estimation is performed in air using the manufacturers software (Nanoscope). Briefly, a square image of rough polycrystalline Ti sample (SI Figure S2 a) is imaged with the tip at 2 nN setpoint. The obtained images are flattened, low pass filtered, and tip estimation is

performed. The tip shape was obtained as shown in SI figure S2 b and the tip end (apex) radius was obtained as 22.89 nm. After the adhesion force measurements, the tip is imaged with the scanning electron microscopy (SEM) to obtain the tip apex radius of 23.06 nm (apex diameter of 46.13 nm, see SI figure S2 c). Accounting for the experimental wear of the tip, the average of the tip radius was estimated to be 22.97 ± 0.08 nm. This value is used throughout the manuscript to calculate the work of adhesion values from the measured adhesion force values.

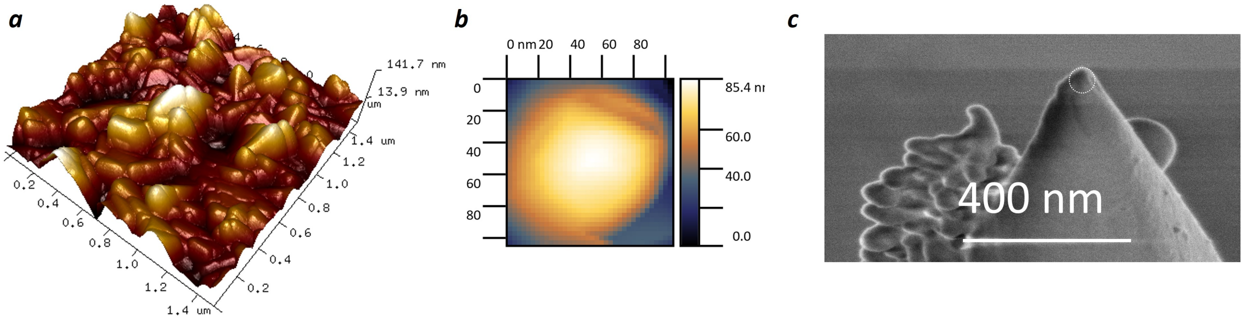

Figure S2: (a) Height image of the Ti sample scanned with the ScanAsyst Fluid tip before experiment. (b) The tip shape estimated from the Nanoscope software. (c) Scanning electron microscope image of the tip after the experiment. Diameter of the apex is shown by the dotted white circle.

3 Estimation of experimental error

The experimental error of adhesion force measurements is estimated by repeated mapping of a single line that consists of 256 pixels (pixel area: 4 nm^2) of the gold electrode in 10 mM Na_2SO_4 electrolyte. The measurement was done under no applied bias voltage to ensure that no electrode processes is interfering with the error estimation. Every pixel was repeated mapped for 12 consecutive times (along y axis of the map) to obtain a corresponding topography (SI figure S3a) and the measured adhesion (SI figure S3b) maps. The average mean adhesion of every measured pixel vs the height of the pixel is plotted in SI figure S3c.

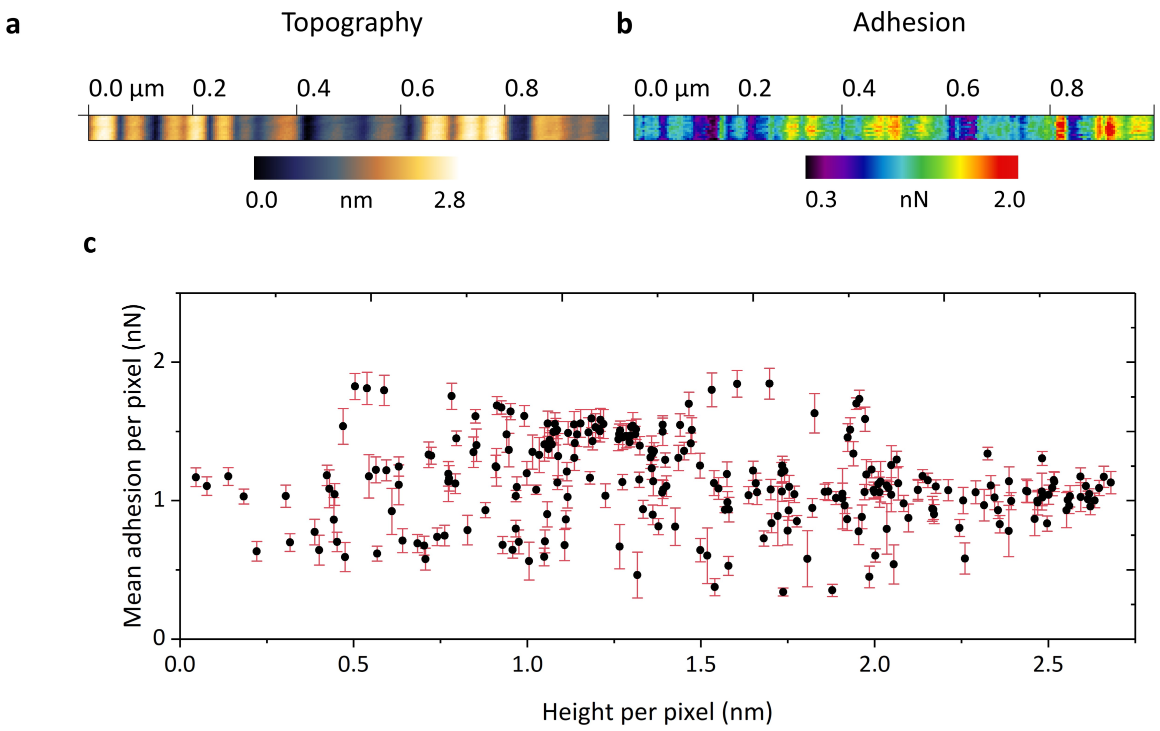

Figure S3: Single line scan of the gold electrode immersed in the 10 mM Na_2SO_4 electrolyte. (a) Topography the repeated line scan and (b) the corresponding adhesion map. (c) The mean height of every pixel (256 pixels) at the line scan vs the corresponding mean adhesion of the pixel repeatedly measured over 12 seconds (at the tip velocity of 256 pixels per second). The error bars correspond to the standard deviation of 12 adhesion measurements per pixel.

The standard deviation distribution of the all measured pixels is shown in the SI figure S4. The center of this distribution (experimental error) is centered around 77pN with the lower and upper bound of 22 pN and 219 pN respectively.

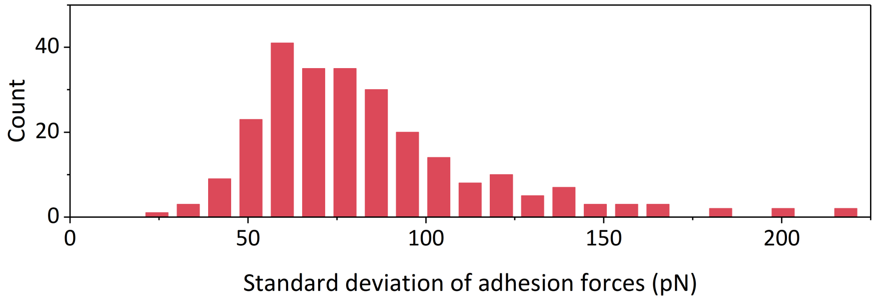

Figure S4: Distribution of standard deviation of adhesion forces of all measured pixels.

4 Topography images at different applied potentials

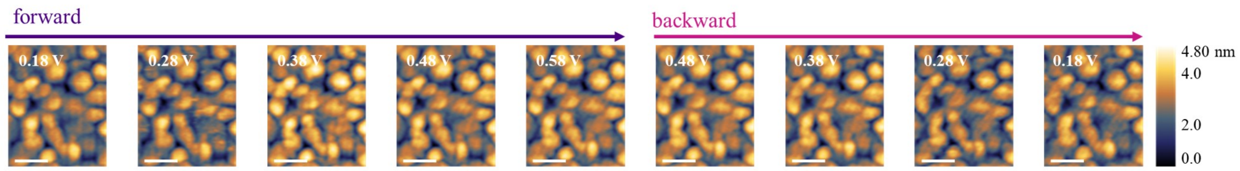

Figure S5: Topography maps of the scan area during the experiment in 10 mM sodium sulphate electrolyte at all different applied potentials during forward and backward scan. Scale :100 nm.

5 Structural characterisation

Upon X-ray diffractogram analysis of the as prepared gold electrodes, we see large peak at around 38.1° corresponding to Au(111) crystal orientation. Upon closer look, we also notice two smaller peaks corresponding to (100)-crystal plane of the titanium adhesion layer.

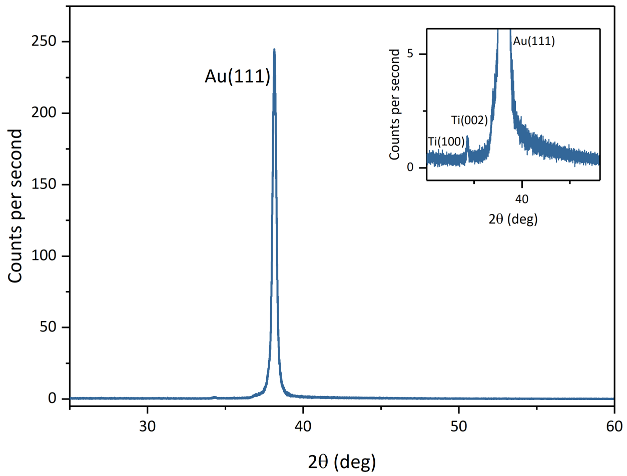

Figure S6: X-ray diffraction patterns of the poly-crystalline gold electrode deposited on top of 5 nm titanium adhesion layer.

6 Cross-section profiles of topography images at three different applied potentials

The cross-section profiles of the topography images are obtained along both AFM scan axis to observe if there are any noticeable changes in the gold grains during three applied potentials: 0.18 V (double layer), 0.58 V (sulphate adsorption) and 0.18 V (double layer). We observe slight increase in the height in a few grains along both scan axis during the sulphate adsorption which reverts back to closer to original values upon the reversal of potential cycling.

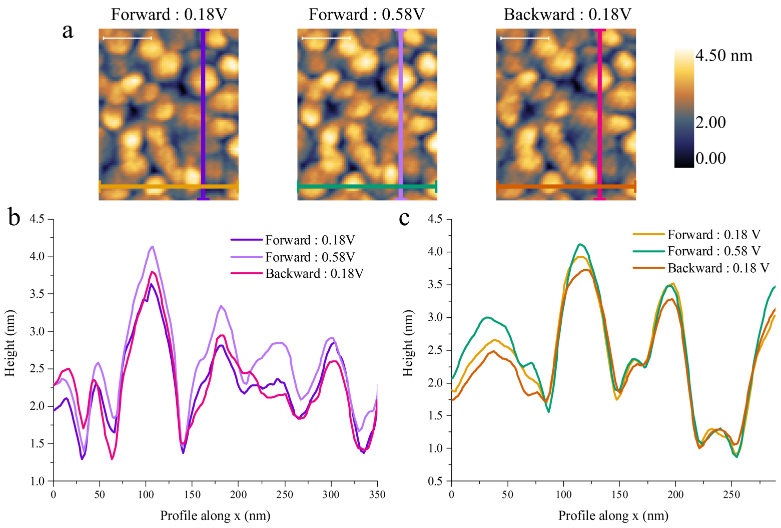

Figure S7: Topography maps of the gold during the experiment in 10 mM sodium sulphate electrolyte (a) during the 0.18 V of the forward scan: first potential step, at 0.58 V during forward scan, during the 0.18 V of the backward scan: last potential step. Extracted line profiles of height along the (b) y-axis and (c)x-axis plotted vs the x-coordinates of the topography maps.

7 Grain identification

The individual grains were identified based on the height segmentation of the grains by using the watershed algorithm[7] on the topography image obtained at the lowest applied potential (0.18 V). The same mask was then applied to the adhesion maps acquired at all applied

potentials. To highlight the adhesion forces mapped on the grains the mask was inverted and applied on the adhesion force maps obtained all applied potentials.

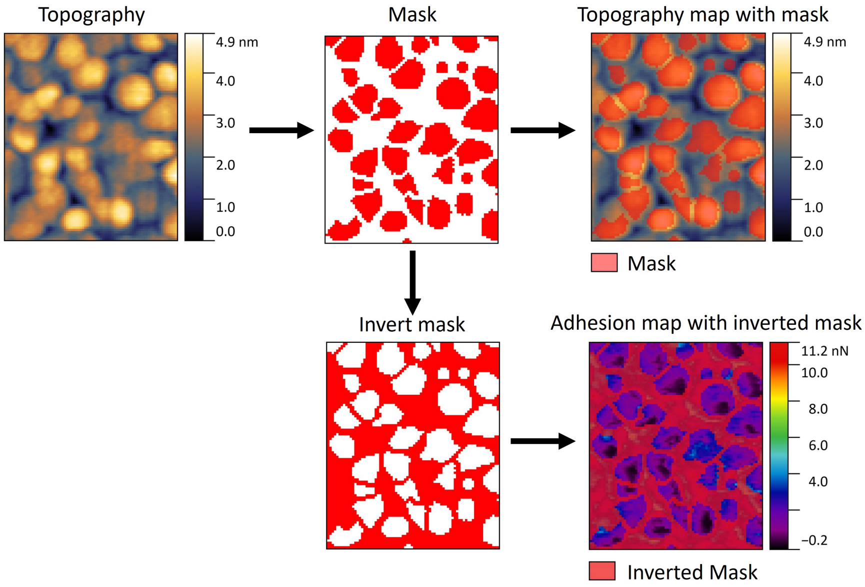

Figure S8: The process of grain identification to perform grain dependent adhesion force analysis. Exposed areas on the adhesion map corresponds to individual grains.

The mask has been used to do several analyses on the grain morphology. Figure S9 represents the statistics on the equivalent grain radius in the in-plane (or lateral) direction (a) and the number of pixels per grain (b). The average grain (lateral) size is around 40 nm, and most grains contain at least 50 pixels.

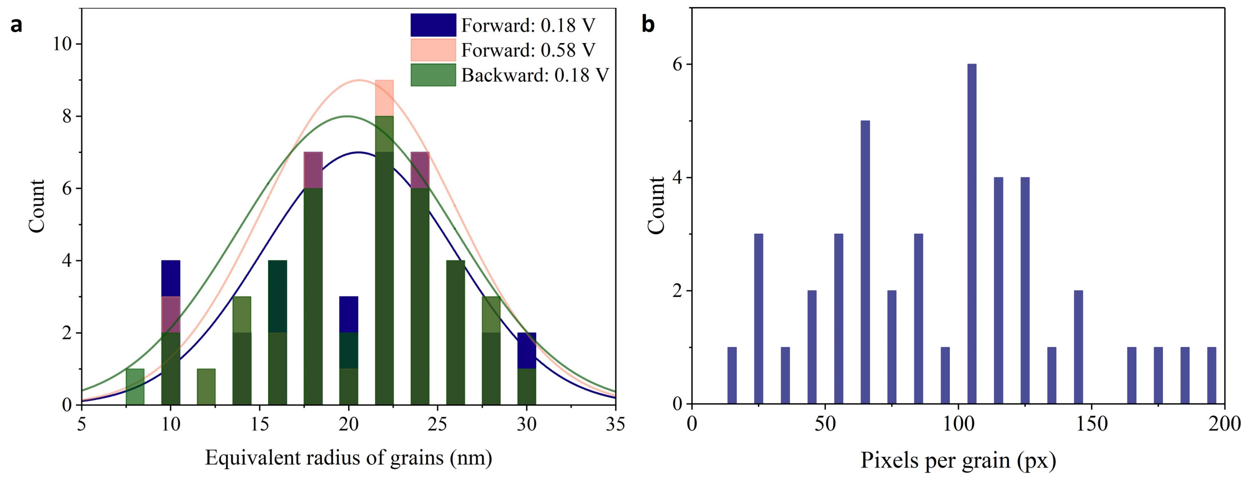

Figure S9: (a) Equivalent (lateral) disc radius (in-plane radius) of marked gold grains at different applied potentials (low: 0.18V - high: 0.58V - low: 0.18V). (b) The distribution of investigated pixels per grain for all the marked grains in the map.

The grain curvature (inverse of curvature radius, given in μm^{-1}) was obtained by fitting the height of the grain with a quadratic polynomial. The negative sign of curvature (also, in our case) indicates a convex grain shape.

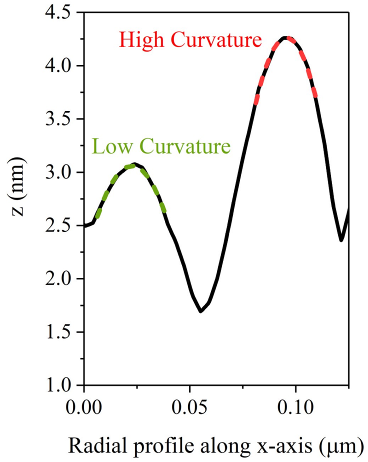

Figure S10: Cross-section of two grains with variable height, fitted with quadratic polynomial. The fits are shown as the dashed lines.

8 Potential dependent adhesion maps with no applied mask

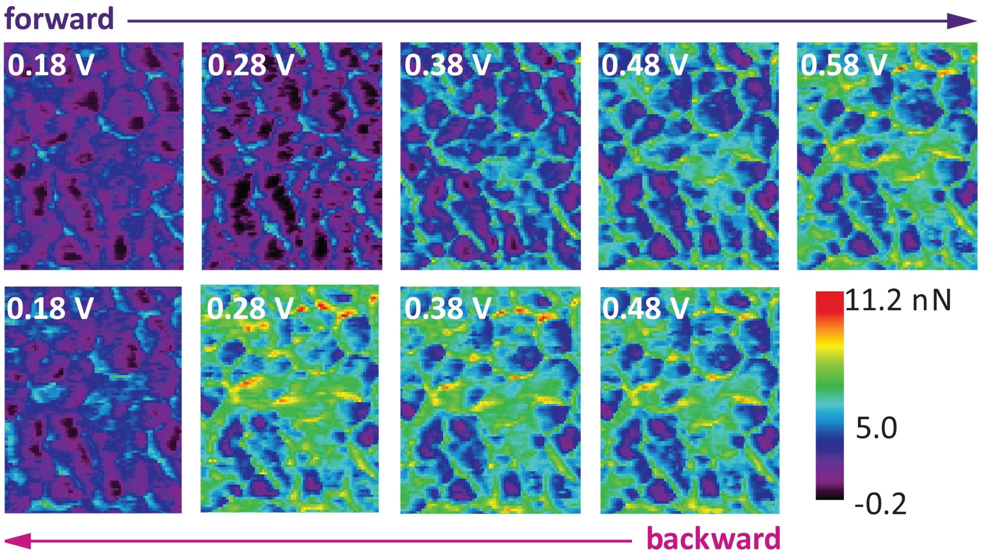

Figure S11: The adhesion maps obtained at different applied potentials without applying an mask overlay.

9 The influence of surface grain curvature on the work of adhesion

We highlight that grains with different grain curvature have different adhesion force responses with bias. However, the surface grain curvature has an influence on the adhesion force measurement (F_{ad}) through equation S1.

$$W_{ad} = \frac{F_{ad} \times (R_s + R_t)}{2 \times \pi \times R_t \times R_s} \tag{S1}$$

where R_s and R_t are the radius of surface grains and the radius of the tip respectively. Note that R_s is not the lateral grain radius but the radius of curvature of the grain, which includes information on the grain height (see Figure S12). The radius of curvature is much larger than the lateral grain radius. Larger out-of-plane radius of curvature compared to the

in-plane grain radius is standard in thin films with good wetting. Wetting and nucleation density determine the in-plane vs out-of-plane curvature anisotropy.[4,5] In this work, the relevant parameter is the out-of-plane curvature, which in the end is the one that is linked to the density of (111)-step terraces and/or exposure to higher order facets. These are both correlated to the density of broken bonds and thus activity.[8]

Figure S13 shows how the work of adhesion changes as a function of adhesion force and grain curvature (i.e. $1/R_s$) as given by the equation above. One can see that curvature has mostly an effect on the adhesion work when the force is large. In this work, grain curvatures are $\leq 10\ \mu m^{-1}$, resulting in a very small correction to the adhesion work. This further concludes that the distinct behaviour of the grains at different applied potentials stem not from the morphology of the grain but from the curvature induced differences in the solid-liquid interface probed by the tip.

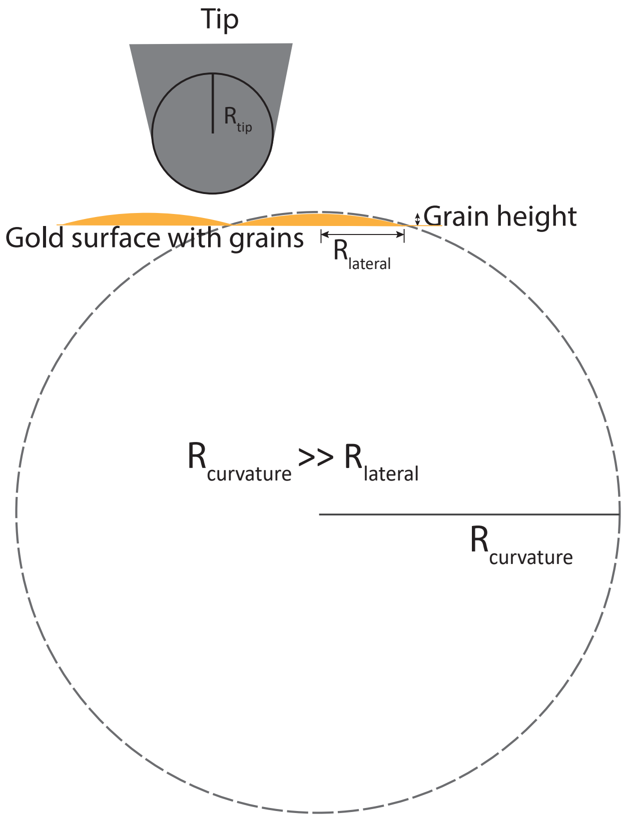

Figure S12: Schematic illustration of tip interacting with the gold grains.

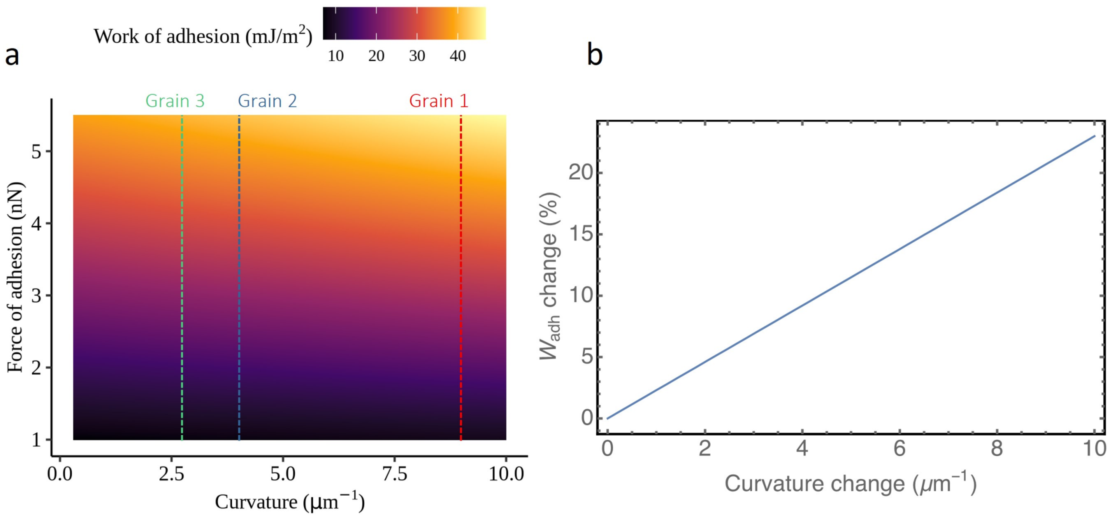

Figure S13: (a)The curvature vs force of adhesion dependence on the calculated work of adhesion. The lines indicate the corresponding curvatures of the three grains marked in figure 3a.(b) Relative change in the work of adhesion(W_{ad}) as a function of change in curvature

10 Potential dependent adhesion forces of the marked grains

The potential dependent mean adhesion forces of the three marked grains (Figure 3a) are shown in Figure S14 a. The uncorrected work of adhesion ($<W_{ad}>$) of the same three grains using the DMT theory of contact mechanics is shown in figure S14 b. This uncorrected $<W_{ad}>$ does not account for the curvature of the gold substrate grains.

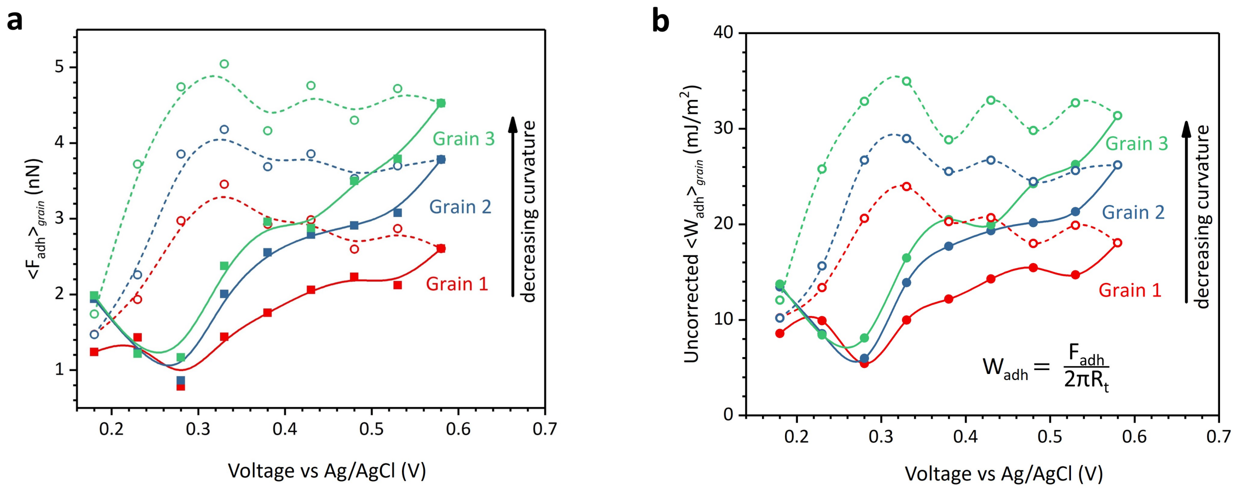

Figure S14: (a) The mean adhesion force of the three marked grains shown in figure 3a at every applied potential. (b) The calculated work of adhesion from the mean adhesion force without accounting for the grain-dependent curvature correction (DMT theory of contact mechanics).

10.0.1 Statistical analysis of grain curvature dependent adhesion forces

Upon inspecting the more grains with different curvatures within the mapped area (figure S15 a) we observe a similar trend of mean grain adhesion forces with respect to the applied potentials. The marked grains were grouped (in 6 groups) based on their curvatures with a maximum standard deviation of $\pm 0.6 \mu m^{-1}$. As the curvature of the grains in these groups decreases the potential dependent adhesion response of the grain increases as expected.

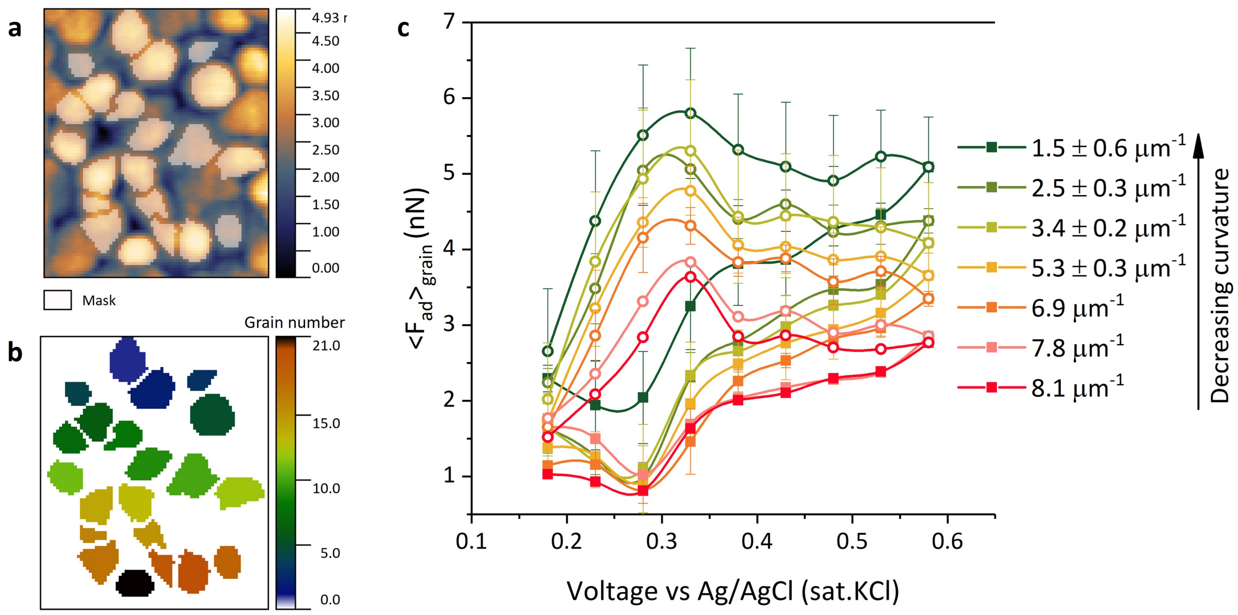

Figure S15: (a) Topography map of the gold electrode mapped during adhesion maps marked with 21 grains under study, (b) Extracted grains with corresponding grain numbers, (c) Mean adhesion of the grains grouped together based on their curvature values. The error bar on the y-axis corresponds to the standard deviation of adhesion values among the grouped grains of similar curvatures.

11 Inclination maps

The software package Gwyddion 2.63 was used to analyse the data set and to enable the masking and overlays. The grain inclination maps (in radian) are obtained from the topography maps of the area under study using the equation S2[1],

$$\theta(x, y) = \tan^{-1} \sqrt{\left(\frac{dz}{dx}\right)^2 + \left(\frac{dz}{dy}\right)^2} \tag{S2}$$

The force curves obtained at every pixel of the data were checked using the native Nanoscope analysis software.

The inclination line profile of the three grains in Figure 2 was obtained from the inclination maps. The cross-section reveals minor differences in inclination for all three grains, thereby eliminating the effects of grain inclination/tip interaction as the reason for distinct

potential-dependent adhesion behavior of the all three grains.

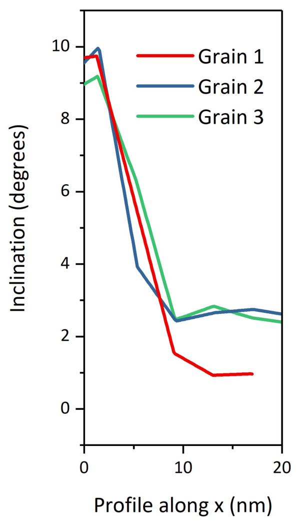

Figure S16: The inclination of three grains (in degree = inclination in radians $\times\frac{180}{\pi}$) shown in figure 3a plotted vs the radial profile.

12 Estimation of pzc by voltammetric approach

The point of zero (pzc) was calculated based on prior works[6,9] which involves application of series of incremental potentials on the electrode of interest to obtain the corresponding current-time responses (i-t curves). The pzc is lies at the potential where the capacitive response at the beginning of the i-t curves change from negative to positive (for anodic potentials) and in our case it was estimated to be around 0.23 V vs Ag/AgCl (sat. KCl).

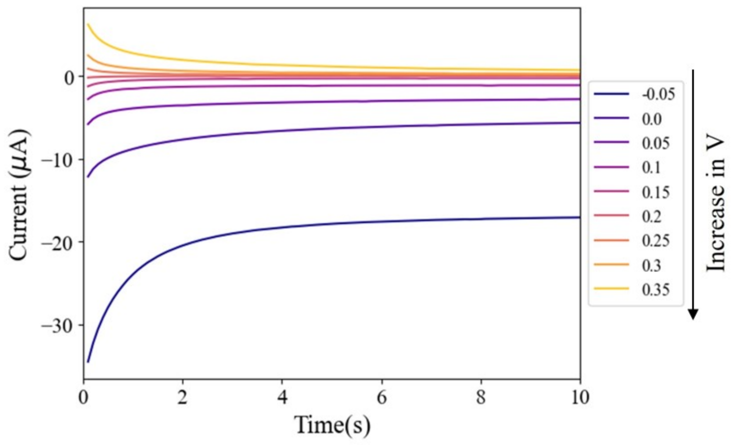

Figure S17: The i-t curves obtained on a fresh gold electrode to calculate the pzc using voltammetric approach. The black arrow indicates the order of applied potentials.

13 Closeup of a single marked grain at three different applied potentials

The evolution of adhesion forces on a single grain (marked via watershed algorithm) at three applied potentials: 0.18 V (double layer), 0.58 V (sulphate adsorption) and 0.18 V (double layer). We observe distinct regions of low and higher adhesion regions on the same grain even at lower potentials (0.18 V). As the potential is increased to 0.58 V these different regions increase in adhesion.

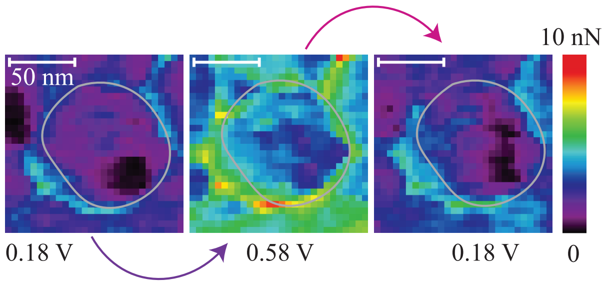

Figure S18: Closeup of same grain area at the lowest, highest and lowest applied potentials. The grain boundary is marked is grey colour.

14 Spatial distribution of GP1 and GP2

Figure S19 highlights the spatial distribution of pixels with high and low adhesion in the mapped area, denoted by GP1 and GP2 respectively. To visualise the spatial distribution of the GP1 and GP2 the adhesion data was colour mapped to a 2-colour scale based on the pixel histogram as shown in Figure S19c. Two distinct adhesion force ranges (2-4 nN and 4-6 nN) are assumed pixels in S19 a and b were marked in blue or red depending on their F_{adh}. For instance, if a pixel F_{adh} value is between 2 and 4 nN, it is marked in red. Figure S19a, shows a 3D projection of topography overlaid with the adhesion data. The grain boundaries of the map are marked in white in Figure S19b to enable clear visualisation of grains and their corresponding adhesion values (at every pixel). This map corresponds to the highest applied potential, where GP1 and GP2 are about 50:50. We can see that some grains appear single color (GP1 or GP2), while some others have areas of each of the two colors (GP1 and GP2).

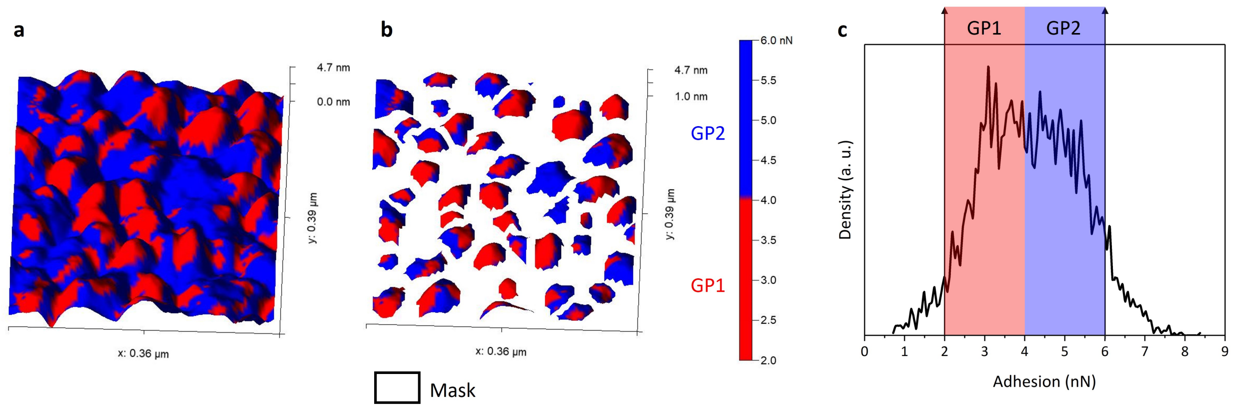

Figure S19: 3D topography at 0.58 V, overlaid with a 2-color adhesion force scale, where blue/red marks pixels with high/low adhesion (GP2/GP1). (a) and (b) are the same data without and with masking of the grain boundary areas, respectively. (c)Adhesion distribution within the marked grains and the adhesion regions correspond to the two grain populations respectively.

References

(1) Mark Aarts, Stefan van Vliet, Roland Bliem, and Esther Alarcon-Llado. Investigation of copper nanoscale electro-crystallization under directed and non-directed electrodeposition from dilute electrolytes. CrystEngComm, 23:3648–3653, 2021.

(2) Jennifer M. Black, Deron Walters, Aleksander Labuda, Guang Feng, Patrick C. Hillesheim, Sheng Dai, Peter T. Cummings, Sergei V. Kalinin, Roger Proksch, and Nina Balke. Bias-dependent molecular-level structure of electrical double layer in ionic liquid on graphite. Nano Letters, 13:5954–5960, 12 2013.

(3) Jennifer M. Black, Mengyang Zhu, Pengfei Zhang, Raymond R. Unocic, Daqiang Guo, M. Baris Okatan, Sheng Dai, Peter T. Cummings, Sergei V. Kalinin, Guang Feng, and Nina Balke. Fundamental aspects of electric double layer force-distance measurements at liquid-solid interfaces using atomic force microscopy. Scientific Reports, 6, 9 2016.

(4) M. J. Rost, D. A. Quist, and J. W. M. Frenken. Grains, growth, and grooving. Phys. Rev. Lett., 91:026101, 2003.

(5) C V Thompson. Grain growth in thin films. Annual Review of Materials Science, 20(1):245–268, 1990.

(6) Yujin Tong, François Lapointe, Martin Thämer, Martin Wolf, and R. Kramer Campen. Hydrophobic water probed experimentally at the gold electrode/aqueous interface. Angewandte Chemie International Edition, 56:4211–4214, 4 2017.

(7) L. Vincent and P. Soille. Watersheds in digital spaces: an efficient algorithm based on immersion simulations. IEEE Transactions on Pattern Analysis and Machine Intelligence, 13(6):583–598, 1991.

(8) Oluwasegun J. Wahab, Minkyung Kang, Enrico Daviddi, Marc Walker, and Patrick R.

Unwin. Screening surface structureelectrochemical activity relationships of copper electrodes under co2 electroreduction conditions. ACS Catalysis, 12(11):6578–6588, 2022.

(9) Yufei Wang, Emma Gordon, and Hang Ren. Mapping the potential of zero charge and electrocatalytic activity of metal-electrolyte interface via a grain-by-grain approach. Analytical Chemistry, 92:2859–2865, 2 2020.
